# Plasma and urine neutrophil gelatinase-associated lipocalin in the diagnosis of new onset acute kidney injury in critically ill patients

**DOI:** 10.1186/cc13958

**Published:** 2014-07-01

**Authors:** Ramprasad Matsa, Emma Ashley, Vivek Sharma, Andrew P Walden, Liza Keating

**Affiliations:** 1 Department of Intensive Care Medicine, University Hospitals North Staffordshire, Stoke-on-Trent ST4 6QG, UK; 2 Department of Biochemistry, Kingston Hospital NHS Trust, Galsworthy Road, Kingston upon Thames KT2 7QB, UK; 3 Department of Anaesthetics, St George’s Healthcare NHS Trust, Blackshaw Road, Tooting, London SW17 0QT, UK; 4 Department of Intensive Care Medicine, Royal Berkshire Hospital NHS Foundation Trust, London Road, Reading, RG1 5AN, UK

## Abstract

**Introduction:**

Neutrophil gelatinase-associated lipocalin (NGAL) has been demonstrated to be a useful early diagnostic biomarker of acute kidney injury (AKI) where the timing of the insult is certain. However, NGAL is not well validated in adult critical care practice because of indeterminate timing of injury. Therefore, we sought to establish the predictive ability of both urine and plasma NGAL to detect AKI in ICU patients.

**Method:**

This prospective observational study was performed in a busy large district general hospital mixed surgical-medical ICU in Reading, UK. Consecutive adult admissions to the ICU, with absence of chronic kidney disease, renal transplant or AKI as defined by RIFLE criteria were included. Blood and urine specimens were collected at admission and every 24 hours until 72 hours and tested for NGAL. The purpose of the study was to assess whether urinary NGAL (uNGAL) or plasma NGAL (pNGAL) can predict the occurrence of AKI at an earlier point of time than the conventional markers, that is creatinine and urine output as is used in RIFLE criteria.

**Results:**

Over a 12-month period, 194 patients were enrolled. In total, 59 (30.4%) patients developed AKI. The admission pNGAL and uNGAL were significantly higher in the patients who developed AKI compared to the non-AKI patients (436 ng/mL (240, 797) versus 168 ng/mL (121.3, 274.3) *P* <0.001 and 342 ng/mL (61.5, 1,280) versus 34.5 ng/mL (11.5, 107.75) *P* <0.001 respectively). Hospital mortality was higher in the AKI group (17% versus 4%). Plasma NGAL performed fairly on admission (AUROC 0.77) and thereafter performance improved at 24 and 48 hours (AUROC 0.88 and 0.87) following ICU admission. Urine NGAL had a fair predictive value on admission (AUROC 0.79) and at 24 hours (AUROC 0.78) and was good at 48 hours (AUROC 0.82).

**Conclusions:**

In critically ill patients without pre-existing kidney disease, both pNGAL and uNGAL measured at admission can predict AKI (defined by RIFLE criteria) occurrence up to 72 hours post-ICU admission and their performance (AUROC) was fair. The accuracy of NGAL appeared to improve slightly as patients progressed through their ICU stay. Serial measurements of NGAL (both pNGAL and uNGAL) may be of added value in an ICU setting to predict the occurrence of AKI.

## Introduction

Acute kidney injury (AKI) is a complex and heterogeneous process and is associated with high morbidity and mortality especially among critically ill patients [1]. The main pathophysiological process is renal tubular ischaemia, which is often multifactorial including sepsis, major surgery, low cardiac output, hypovolaemia and medication toxicity. Such insults frequently occur throughout a patient’s stay on the intensive care unit (ICU) [1,2].

Consensus diagnosis of AKI depends upon the detection of oliguria and/or a rise of serum creatinine level [3]. However, changes in creatinine often lag behind reductions in the glomerular filtration rate (GFR) questioning its utility as a biomarker to pre-empt deterioration in renal function [4]. The Acute Dialysis Quality Initiative (ADQI) consensus has recommended RIFLE criteria to stratify three severity stages and two outcome stages for AKI - risk, injury, failure, loss and end-stage renal failure; relying on creatinine and urine output for their definition [3]. Despite advances in understanding the mechanisms of AKI the morbidity and mortality, especially in ICU patients, has not changed for some years. One key factor identified is a delay in diagnosis [5,6] suggesting a need for more accurate and timely biomarkers. This would allow earlier identification of patients who may benefit from enhanced resuscitative care and pre-emptive renal support to prevent further renal injury.

Neutrophil gelatinase-associated lipocalin (NGAL) has been shown in multiple laboratory experiments to be secreted early in the development of AKI. This has been confirmed in several clinical settings including cardiac surgery, following contrast administration, in the emergency department and in paediatric intensive care [7-9].

To the best of our knowledge, only a few studies have examined the role of NGAL to predict AKI occurrence in a general adult ICU [10-15]. A recent systematic review showed variability in the predictive ability of NGAL [16] related to inconsistencies in study design, patient inclusion and method of measurement. Most of these studies have included patients on admission who have pre-existing renal disease, (acute or chronic) leading to uncertainty about the timing and type of renal insult. It is also unclear whether urinary NGAL (uNGAL) or plasma NGAL (pNGAL) provide the best predictive ability. This study was designed to examine the predictive ability of both pNGAL and uNGAL in a heterogeneous adult ICU population with no known pre-existing renal disease prior to admission.

## Materials and methods

### Study design and setting

This prospective observational study was conducted in a mixed medical and surgical adult ICU. All consecutive patients admitted from May 2011 until April 2012 were screened. Patients were enrolled within their first 24 hours of admission. The patient’s consent or the assent from next of kin (if the patient was unable to consent) was obtained by the trained staff prior to the participation in this study. Only patients who offered their consent were included in the study. If the patient lost their capacity (having consented earlier), their original decision was upheld. If a patient who was initially unable to consent (and assent from the next of kin was obtained) decided to withdraw from the study at a later stage, their wishes were respected and they were excluded from the study. The study was approved by the National Research Ethics Committee (Reference: Central London REC 3. 11/H0716/9) and by the Department of Research and Development, Royal Berkshire Hospital NHS Foundation Trust.

### Inclusion and exclusion criteria

Consecutive adult (>18 years) patients admitted to the ICU were screened for inclusion. Exclusion criteria were refused consent, end-stage renal disease (ESRD), previous renal transplant, patients already on renal replacement therapy (RRT), patients referred to the ICU for RRT and patients with AKI as defined by RIFLE criteria for risk, injury or failure.

### Definition of acute kidney injury

We used the urine output and creatinine components of the RIFLE criteria to define AKI [3]. The baseline creatinine was the lowest value in the previous three months or, when this was unknown, we estimated the GFR from the modification of diet in renal disease (MDRD) formula and back-calculated the creatinine [17]. The criteria were used to exclude patients on admission with pre-existing renal injury and were recorded daily in all patients for the first 72 hours of ICU stay and at discharge from the ICU.

### Clinical and laboratory data collection

Data was collected daily on a case report form (CRF) for each patient. Data on demographics, medical history and admission diagnosis were collected in addition to daily physiological variables, need for interventions such as RRT and exposure to potential nephrotoxic compounds (including contrast media and medications). Standard laboratory blood tests were performed daily as part of usual clinical care. Urine and blood samples for NGAL were drawn on admission, at 24, 48 and 72 hours. The reason for not collecting samples in such time periods was discharge and/or death of the patients.

### Sample processing and measurement for NGAL

The blood and urine samples were sent to our clinical biochemistry laboratory and processed within 48 hours. The blood samples were centrifuged at 3,200g for 10 minutes and 2 mL aliquots of supernatant were stored at -70°C for batch analysis. Urine samples were centrifuged at 4,000 g for 5 minutes and 5 mL aliquots of supernatant were stored at -70°C. The samples were assayed for NGAL using Bioporto™ Diagnostics turbidimetric assay on the Roche-Cobas c501 clinical chemistry analyser (Roche, Basel, Switzerland). Laboratory investigators were blinded to the clinical information throughout the study. In order to validate the assay, the manufacturer’s performance claims were compared with data collected in the laboratory. The method was evaluated in accordance with the Association of Clinical Biochemistry document ‘Measurement verification in the clinical laboratory’ [18].

### Statistical analysis

Means and standard deviations are used to describe continuous variables. For heavily skewed distributions, the median and interquartile range was used instead. Categorical variables are expressed as proportions.

Receiver operator characteristic (ROC) analysis [19] was used to explore the ability of NGAL to predict AKI within 72 hours when measured on admission. ROC curves are presented and the area under the curve (AUC) has been calculated. Sensitivity and specificity, positive and negative likelihood ratios, and positive and negative predictive values are reported for a wide range of thresholds, including the threshold that maximises combined sensitivity and specificity (Youden’s index) and the manufacturer recommended threshold (400 ng/ml for plasma and 350 ng/ml for urine). The ability of NGAL measured at 24 hours and 48 hours to predict subsequent AKI was explored to evaluate the benefit of obtaining serial NGAL measurements over time. Detailed statistical methods are provided in Additional file 1.

## Results

### Patient demographics

**Figure 1:**
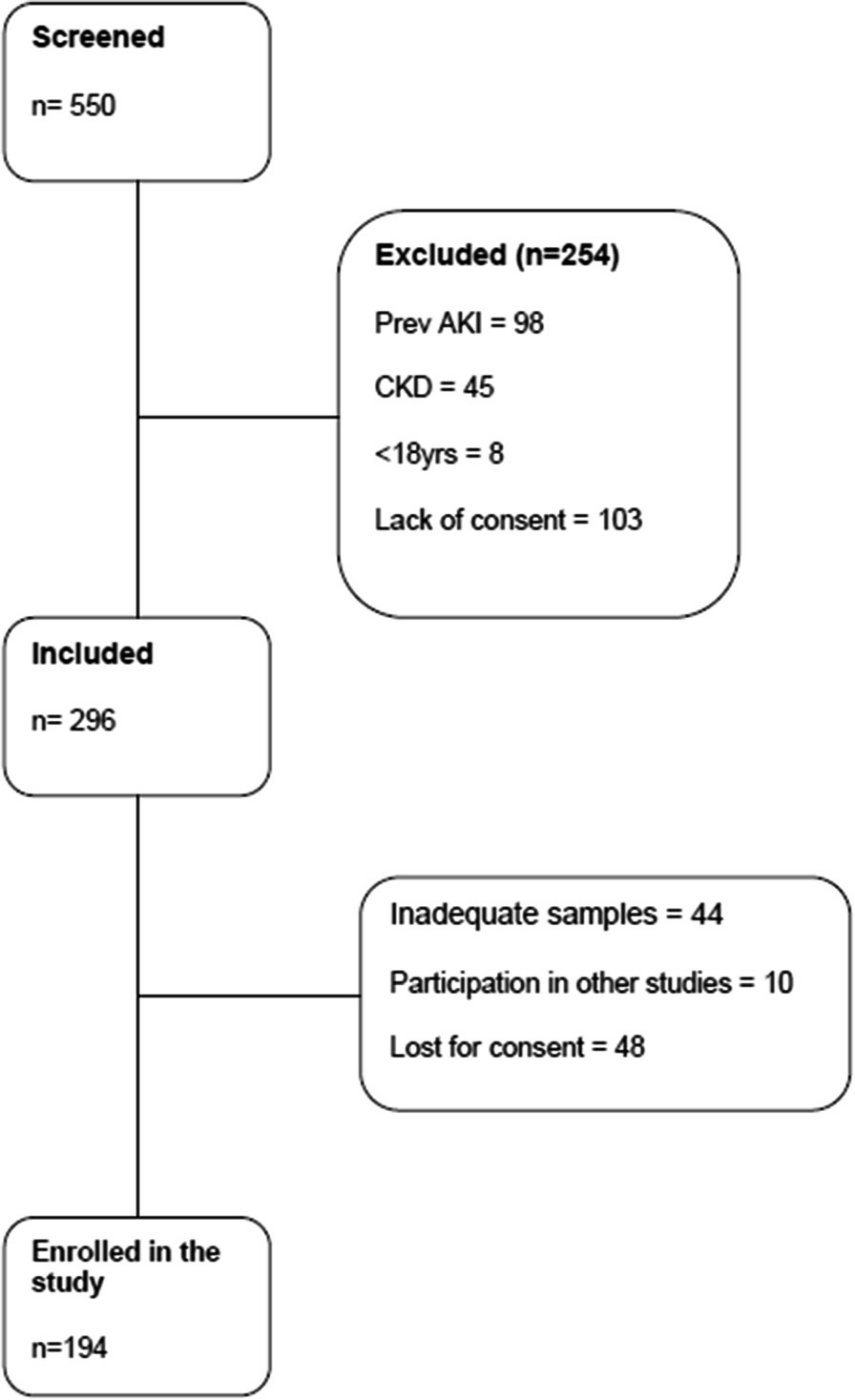
Flow chart of patient inclusion into the study.

The flow chart depicting the patient enrolment is described in Figure 1. Five hundred and fifty consecutive patients were screened between May 2011 and April 2012, with 194 enrolled in the study. We measured serial pNGAL and uNGAL levels on admission and at every 24, 48 and 72 hours. However, the number of patients for whom the NGAL was measured decreased with time after admission to ICU as they were either discharged, died and/or samples were not available (Table 1).

**Table 1:** Summary of results of serial measurements of both plasma and urinary NGAL to predict AKI at different time points

	**Number of patients**	**Threshold values (ng/mL)**	**Sensitivity (95% CI)**	**Specificity (95% CI)**	***NPV***	***AUROC***
pNGAL at 0h for AKI occurrence at 24, 48 or 72 h	187	400	0.6 (0.43-0.74)	0.85 (0.77-0.91)	0.86	0.7668
pNGAL at 24h for AKI occurrence at 48 or 72 h	125	400	0.79 (0.54-0.94)	0.75 (0.64 -0.81)	0.93	0.8807
pNGAL at 48h for AKI occurrence at 72 h	77	400	0.73 (0.30-0.94)	0.95 (0.84-0.99)	0.92	0.8723
uNGAL at 0h hours for AKI occurrence at 24, 48 or 72 h	163	350	0.58 (0.39-0.75)	0.84 (0.76-0.90)	0.87	0.7914
uNGAL at 24h for AKI occurrence at 48 or 72 h	118	350	0.75 (0.48- 0.93)	0.82 (0.71-0.9)	0.94	0.7768
uNGAL at 48h for AKI occurrence at 48 or 72 h	68	350	0.75 (0.35-0.97)	0.88 (0.72- 0.97)	0.93	0.8220

NGAL, neutrophil gelatinase-associated lipocalin; AKI, acute kidney injury; CI, confidence interval; NPV, negative predictive value; AUROC, area under the receiver operator characteristic curve; pNGAL, plasma neutrophil gelatinase-associated lipocalin; uNGAL, urine neutrophil gelatinase-associated lipocalin.

Fifty-nine patients (30.4%) developed AKI based on RIFLE criteria and 42 patients (71.2%) developed AKI within the first 24 hours of admission to the ICU. Of these patients, 37% (n = 22) were medical and 63% (n = 37) were surgical. The mean age group of the patients who developed AKI was higher than that of the non-AKI group (62.2 ± 15.71 versus 59.18 ± 15.05). There was no significant difference in the urine output between both the groups (*P* = 0.098). The mean acute physiology and chronic health evaluation II (APACHE II) scores among patients with AKI were significantly higher than the group with no AKI (16.69 ± 5.16 versus 12.8 ± 5.3). The AKI cohort was more likely to have been exposed to nephrotoxic medications and radio-contrast dye (29% vs. 17%, *P* = 0.015). Table 2 describes the patient characteristics.

**Table 2:** Demographic and case mix data for all patients

	**All patients**	**No AKI**	**AKI**	***P *** **value**
	**Mean ± sd**	**Mean ± sd**	**Mean ± sd**	
Age (years)	60.1 ± 15.3	59.2 ± 15.1	62.2 ± 15.7	0.214
Female sex	86 (44%)	59 (44%)	27 (46%)	0.791
Weight (kg)	66.6 ± 11.0	66.8 ± 11.4	66.0 ± 10.2	0.619
***Specialty code prior to unit admission *** **( *% *)**				
Accident and Emergency	22 (11%)	13 (10%)	9 (15%)	0.05
Medicine	50 (26%)	38 (28%)	12 (20%)	
Surgery	76 (39%)	46 (34%)	30 (51%)	
Trauma	12 (6.2%)	11 (8%)	1 (2%)	
Other	34 (17.5%)	27 (20.0%)	7 (11.9%)	
***ICU admission***				
APACHE II	14.0 ± 5.5	12.8 ± 5.3	16.7 ± 5.2	<0.001
Creatinine (mg/dL)	80.8 ± 29.1	68.2 ± 18.9	109.4 ± 28.0	<0.001
pNGAL on admission (ng/L)	209 (141, 389)	168.0 (121.3, 274.3)	436.0 (240.0, 797.0)	<0.001
uNGAL on admission (ng/L)	53 (16, 283)	34.50 (11.5, 107.75)	342.00 (61.5, 1,280)	<0.001
Urine output (mL/hour)	67 (40, 100)	70 (45, 100)	60 (36, 90)	0.098
MAP (mmHg)	78.6 ± 16.7	80.54 ± 16.78	74.12 ± 15.82	0.012
Mechanical ventilation (%)	84 (43%)	58 (43%)	26 (44%)	0.886
Vasopressors (%)	54 (28%)	29 (21%)	25 (42%)	0.003
Sepsis (%)	15 (8%)	7 (5%)	8 (14%)	0.136
RRT in first 24 hours (%)	2 (1%)	0 (0%)	2 (3%)	0.093
***Nephrotoxic agents *** **( *% *)**				-
Radio contrast dye	36 (19%)	19 (14%)	17 (29%)	
ACE inhibitor	55 (28%)	37 (27%)	18 (31%)	
Diuretics	37 (19%)	22 (16%)	15 (25%)	
ARBs	10 (5%)	5 (4%)	5 (8%)	
NSAIDs	9 (5%)	7 (5%)	2 (3%)	
Cyclosporin	1 (1%)	0	1 (2%)	
None	104 (54%)	76 (56%)	28 (47%)	
***Outcome***				
Hospital mortality (%)	15 (8%)	5 (4%)	10 (17%)	0.001

AKI, acute kidney injury; sd, standard deviation; ICU, intensive care unit; APACHE II, acute physiology and chronic health evaluation II; pNGAL, plasma neutrophil gelatinase-associated lipocalin; uNGAL, urine neutrophil gelatinase-associated lipocalin; MAP, mean arterial pressure; RRT, renal replacement therapy; ACE inhibitor, angiotensin-converting enzyme inhibitor; ARBs, angiotensin II receptor blockers; NSAIDs, non-steroidal anti-inflammatory drugs.

**Figure 2:**
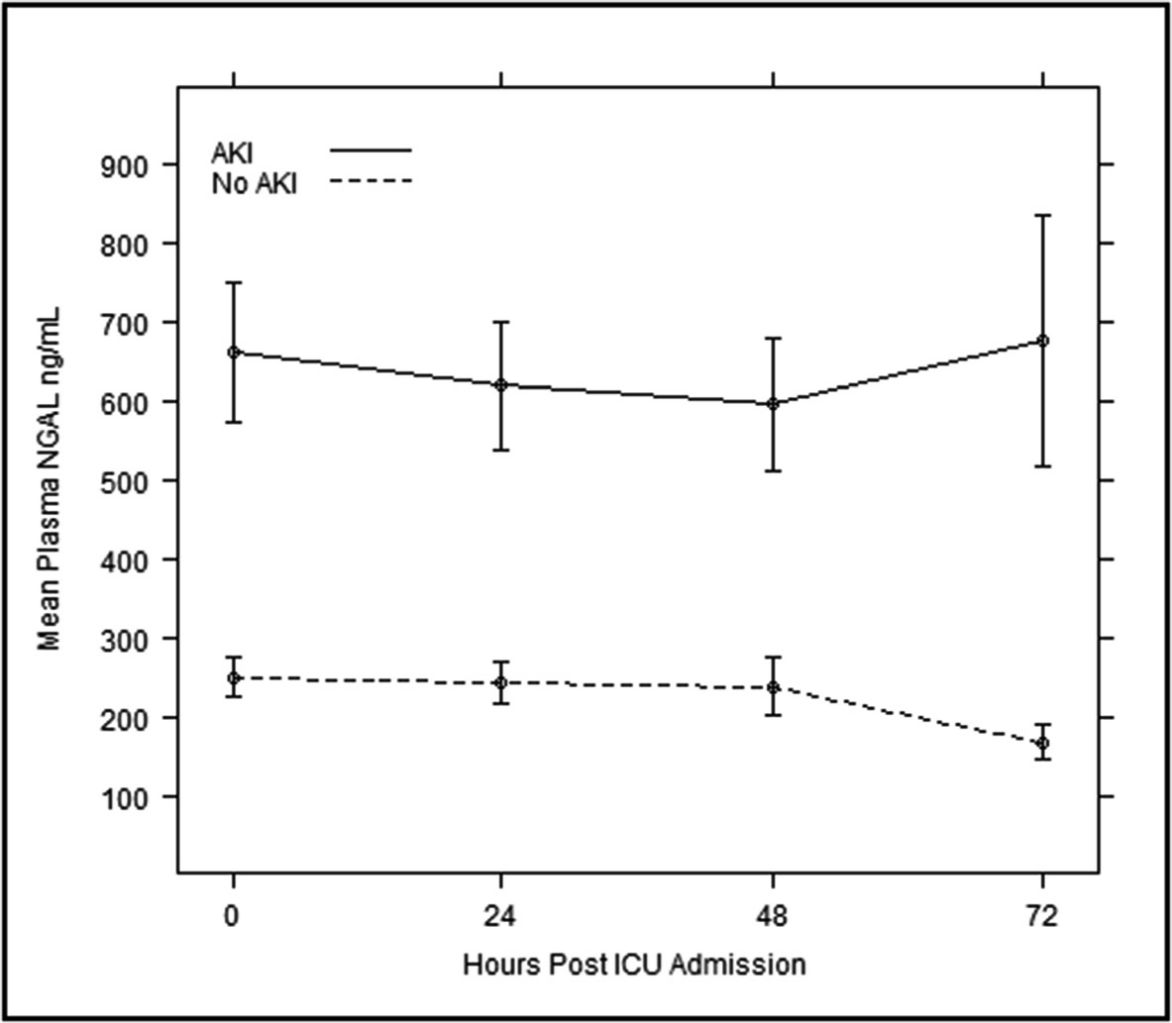
**Serial mean plasma NGAL measurements in those with and without AKI.** AKI, acute kidney injury; NGAL, neutrophil gelatinase-associated lipocalin.

**Figure 3:**
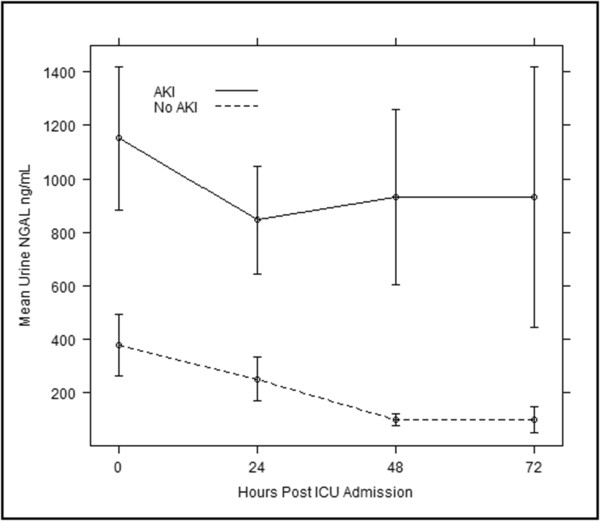
**Serial mean urine NGAL measurements in those with and without AKI.** AKI, acute kidney injury; NGAL, neutrophil gelatinase-associated lipocalin.

**Figure 4:**
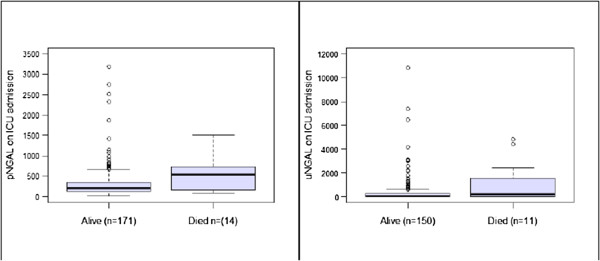
**Plasma and urine NGAL measurements in patients who are alive and dead.** NGAL, neutrophil gelatinase-associated lipocalin.

The pNGAL and uNGAL concentrations both at the time of ICU admission and at any given point of time were significantly higher in AKI compared to that of the non-AKI patients (Figures 2 and 3). The pNGAL gradually increases with time whereas the uNGAL plateaus in patients with AKI. Two patients among the group with AKI required RRT. Mortality rate was higher among the patients with AKI when compared to the patient group with no AKI (17% vs. 4%, *P* = 0.001) although the overall mortality was low (n = 15). Both pNGAL and uNGAL levels were significantly higher in the patients who died (pNGAL: 207 ng/mL (IQR 136 to 356) vs. 538 ng/mL (IQR 165 to 735) and uNGAL: 52.5 ng/mL (IQR 16 to 248) vs. 190 (IQR 21 to 2144) respectively) (Figure 4).

### Plasma NGAL

**Figure 5:**
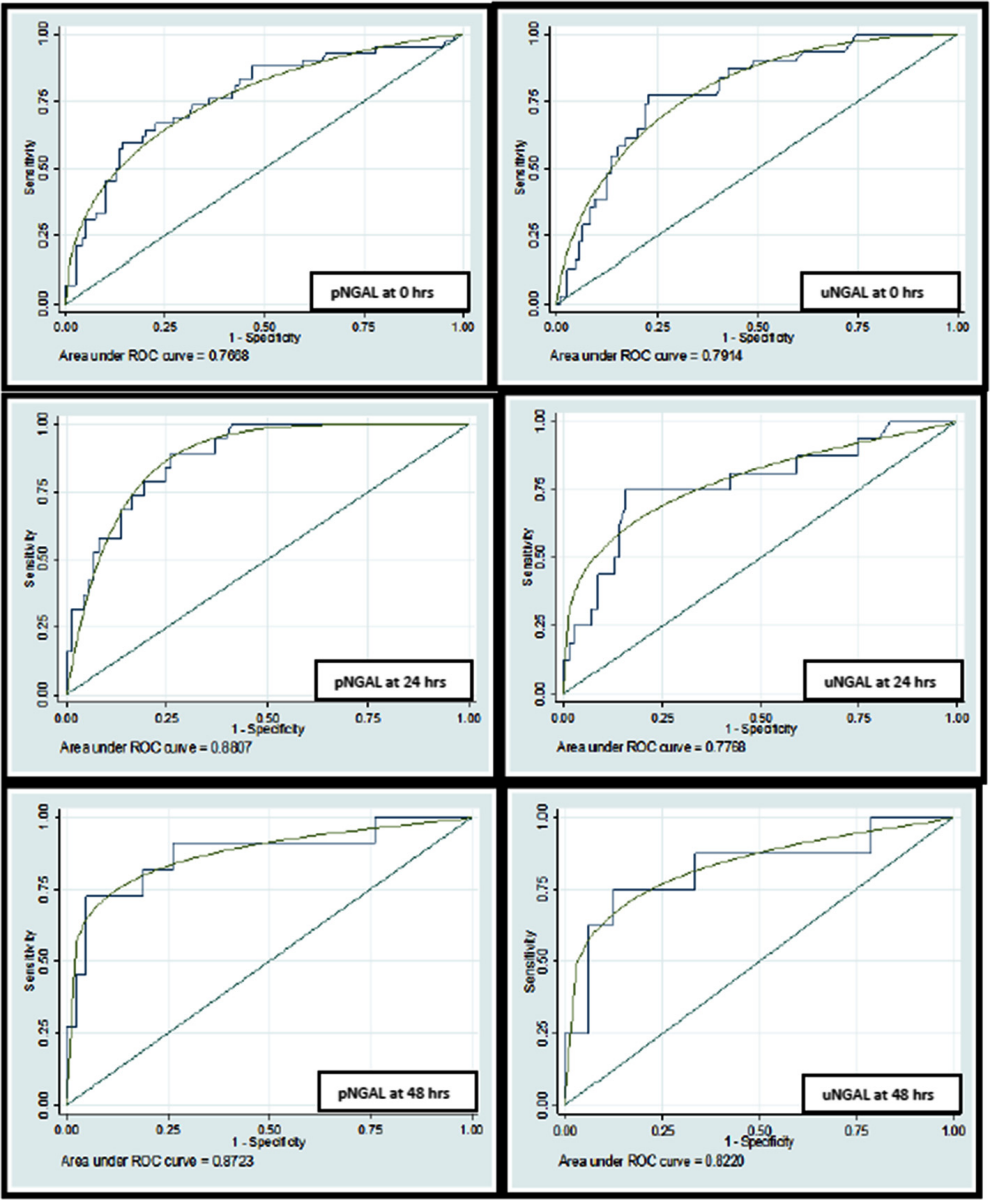
**AUROC curves demonstrating the predictive ability of plasma and urine NGAL and urine NGAL at 0, 24 and 48 hours to predict AKI up to 72 hours.** AKI, acute kidney injury; AUROC, area under the receiver operator characteristic curve; NGAL, neutrophil gelatinase-associated lipocalin.

The accuracy of pNGAL, measured on admission, to predict the occurrence of AKI within 72 hours was assessed. The area under the ROC curve was 0.767 (Figure 5) and, at the manufacturer recommended cut-point of 400 ng/mL, had a sensitivity of 0.60 (95% confidence interval (CI): 0.43 to 0.74) (Table 1) and a specificity of 0.85 (95% CI: 0.77 to 0.91).

We measured serial NGAL measurements to determine utility in admitted patients. The pNGAL measured at 24 hours to predict the occurrence of AKI at 48 and 72 hours of admission to ICU for a given threshold of 400 ng/mL had a sensitivity of 0.79 (95% CI 0.54 to 0.94) and specificity of 0.75 (95% CI 0.64 to 0.85) and the performance was good (area under the receiver operator characteristic curve (AUROC) 0.88) (Figure 5). Similarly the pNGAL measured at 48 hours to predict the occurrence of AKI at 72 hours performed fairly (sensitivity of 0.73 (95% CI 0.39 to 0.94); specificity of 0.83 (95% CI 0.69 to 0.93); AUROC 0.87) (Table 1).

### Urine NGAL

The predictive value of uNGAL (Table 1) measured at admission, at a threshold of 350 ng/mL to predict the occurrence of AKI within 72 hours of ICU admission based on the serum creatinine values and urine output measured at 24, 48 or 72 hours had a sensitivity of 0.58 (95% CI 0.39 to 0.75) and specificity of 0.84 (95% CI 0.76 to 0.90) and the performance was fair (AUROC 0.7914) (Figure 5).

The uNGAL measured at 24 hours to predict the occurrence of AKI at 48 and 72 hours of admission to ICU had a sensitivity and specificity of 0.75 (95% CI 0.48 to 0.93) and 0.82 (0.71 to 0.90) respectively and the performance was fair (AUROC 0.776). The uNGAL measured at 48 hours to predict the occurrence of AKI at 72 hours had a sensitivity of 0.75 (95% CI 0.35 to 0.97) and specificity of 0.85 (95% CI 0.68 to 0.95) and the performance was good (AUROC 0.822) (Table 1).

## Discussion

Neutrophil gelatinase-associated lipocalin has been used for the diagnosis, prognosis and severity assessment of AKI and has been validated in paediatric populations (where comorbidity is low) and in conditions where the timing of the insult is clear (that is cardiac surgery and so on) [20-24]. Its role in an adult ICU population has not been well validated due to the heterogeneity and uncertainty of the timing of the insult. The pathophysiology and causes of AKI in the ICU could be indigenous [25] and may differ from pre-ICU causes (low-volume state, inotropes, contrast injury and so on). This has led to problems in the design and interpretation of studies in the general adult ICU patient cohort. In this study, we sought to overcome these problems by excluding patients with pre-existing chronic kidney disease (CKD) and/or AKI and by looking at the predictive value of both urinary and plasma NGAL at different time points following admission.

We found the incidence of AKI was 30.4% (n = 59) with 71% (n = 42) developing it within the first 24 hours. This incidence correlated well with that anticipated from our sample size calculation but was lower than that found in previous studies due to the exclusion of those patients with pre-existing renal disease [11,12,26]. Both admission pNGAL and uNGAL were found to fairly predict the occurrence of AKI at 72 hours following ICU admission (AUROC 0.766 and 0.791 respectively). Our study also showed that both pNGAL and uNGAL concentrations, at the time of ICU admission and at any given point of time, were significantly higher in AKI compared to that of the non-AKI patients (Figures 2 and 3). de Geus *et al*. [10] found that the performance of pNGAL at admission to predict the AKI severity to be similar to our study [10]. However, utilising admission NGAL values to predict AKI up to seven days can be associated with certain confounding factors. Raised NGAL at admission may correlate tubular ischemia at that point of time. The possibility of a second or multiple insults after the initial insult has been reversed is common in an ICU setting. Therefore, prediction of AKI at a later point of time may not represent true index performance. We have overcome this issue by serial measurements of NGAL. Moreover the study by de Geus included patients with previous renal disease. This will affect the sensitivity and specificity of the test. Another study [11] showed that performance of pNGAL to predict AKI occurrence up to seven days was excellent (AUROC 0.92). The small sample (n = 88) may over-inflate the diagnostic accuracy. Cruz *et al.*[13] found that the predictive value of admission pNGAL to diagnose AKI up to 48 hours was fair (AUROC 0.78) but when extended to five days, pNGAL performance was poor (AUROC 0.67). This suggests that there is a wide variation of NGAL assay performance when prediction is extended beyond five days. Of note, a recent study [27] looking at admission pNGAL to predict AKI occurrence up to 72 hours found results not dissimilar to our study (AUROC 0.78), however, by excluding patients with pre-existing kidney disease we have determined the diagnostic accuracy more precisely.

Many of the preliminary studies performed to evaluate the diagnostic value of NGAL were done using urine analysis yielding promising results, however, these studies were done in a paediatric, non-ICU population, and where time of injury was clearly defined [9,20-23]. Siew *et al.*[15] found that the admission uNGAL values to predict AKI at 24 hours among ICU patients was fair (AUROC 0.71) but did not perform well when extended to 48 hours (AUROC 0.64). The study used a convenience sample from a large biomarker study in acute lung injury and also included patients with previous and existing kidney injury.

Another study performed by Makris *et al.*[28] showed that the uNGAL performance to predict AKI up to five days was excellent (AUROC 0.97). The study mostly included trauma patients. The generalisability of this study, however, is limited due to the small sample size (n = 31). Other ICU studies investigating uNGAL to predict AKI occurrence up to five days or more have shown variable results [10,11,26]. Under normal circumstances, the filtered NGAL is completely absorbed by the proximal convoluted tubule (PCT) and therefore there should be minimal or no NGAL in the urine [29]. If there is damage to the PCT, NGAL is not absorbed and accounts for the rise in NGAL levels in urine. Further evidence suggests that the NGAL production can arise from the injured distal nephron [8]. This could explain the persistent rise in uNGAL in patients with ongoing injury. When the GFR is reduced, serum creatinine increases. Similarly, systemic NGAL (pNGAL) levels increase as NGAL filtration is decreased.

Our study has looked at the occurrence of AKI up to 72 hours. By taking serial samples we were able to show that both uNGAL and pNGAL continue to have fair to good predictive ability (Table 1) in admitted patients suggesting there may be a role in the daily detection of renal injury. The 72-hour time frame is a reasonable time for the pNGAL to accumulate. Of note, the kinetics of pNGAL and uNGAL in our study suggest pNGAL gradually increases with time whereas the uNGAL reaches a plateau (Figures 2 and 3). However, this finding is clearly limited by the small number of samples at 48 hours.

A small study in a selected group of patients with septic shock [12] showed that uNGAL performed better (AUC 0.86) than the pNGAL (0.67) to predict the AKI occurrence 12 hours ahead of time. Therefore, pNGAL may not be a good predictor if the prediction time is short as it may take some time to accumulate and uNGAL may be a better marker in triage situations. Therefore, uNGAL could be a better predictor in conditions where AKI is anticipated that is radio-contrast-induced AKI, coronary artery bypass graft (CABG) and so on. However, one drawback is that sometimes in critically unwell patients, obtaining urine may be difficult and sometimes uNGAL may be elevated with urinary tract infection [14].

The threshold values we used were high compared to similar studies. The difference may be secondary to the type of assays and platform used. We chose an immunoturbidimetric assay due to the fast assay time (10 minutes), a platform compatible with most analysers available in UK laboratories [30,31] and a wide measuring range (25 to 5000 ng/mL) that will allow easy validation in clinical practice.

Our study makes an important contribution to the current body of literature on NGAL and AKI. To the best of our knowledge, this is the first study to prospectively evaluate both plasma and urine NGAL in adult ICU patients where pre-existing renal disease has been excluded. Our study has certain important strengths. First, this is the first published UK study on NGAL to predict AKI in ICU patients; second, our strict exclusion criterion excluded pre-existing kidney disease; third, we powered our study to ensure statistical robustness; fourth, we demonstrated the value of serial sampling of pNGAL and uNGAL; finally, we used a standardised user-friendly platform, reducing laboratory measurement error and allowing other investigators to validate our findings.

Our study also has deficiencies. This is a single-centre study and hence care should be exercised when generalising the results. Although we excluded patients with AKI prior to admission, there may be a small number of patients who might have had an undetected renal insult in the few hours prior to ICU admission. This is an important limitation in any study of early biomarkers in an unselected ICU population. Second, we did not perform a sensitivity analysis to discount comorbidities that could account for the changes in NGAL. Third, there had been a decrease in the number of patients for whom the serial NGAL levels were measured with time (that is 0, 24 and 48 hours). In a general ICU setting this attrition is unavoidable. In some cases, the samples were not available from the patients due to anuresis and/or in the process of end-of-life care. Fourth, urine NGAL levels were not corrected for urinary creatinine concentration to account for possible dilution of urine. Finally, we were unable to correlate NGAL values with clinical outcomes such as mortality and need for RRT due to the small numbers in these groups.

## Conclusions

Our study showed that both pNGAL and uNGAL levels measured at admission to ICU can predict an occurrence of AKI for up to 72 hours of ICU stay. The performance as determined by AUROC is fair. This performance was sustained when measured at serial time points throughout the ICU stay.

## Key messages

• In patients with no kidney disease prior to admission to ICU, both pNGAL and uNGAL have a fair predictive value to diagnose the occurrence of AKI for up to 72 hours.

• This performance is sustained when measured at serial time points throughout the ICU stay.

## Abbreviations

ADQI: Acute Dialysis Quality Initiative; AKI: acute kidney injury; APACHE II: acute physiology and chronic health evaluation II; AUROC: area under the receiver operator characteristic curve; CABG: coronary artery bypass graft; CI: confidence interval; CKD: chronic kidney disease; CRF: case report form; ESRD: end-stage renal disease; GFR: glomerular filtration rate; ICU: intensive care unit; MDRD: modification of diet in renal disease; NGAL: neutrophil gelatinase-associated lipocalin; PCT: proximal convoluted tubule; pNGAL: plasma neutrophil gelatinase-associated lipocalin; RIFLE: risk, injury, failure, loss and end-stage renal failure; RRT: renal replacement therapy; UK: United Kingdom; uNGAL: urine neutrophil gelatinase-associated lipocalin.

## Competing interests

The authors declare that they have no competing interest.

## Authors’ contributions

RM contributed substantially towards the conception and design, funding application, drafting the manuscript, acquisition of data, analysis and interpretation of data, drafting and critically revising it for intellectual content and final approval of the version to be published. EA carried out the biochemical assays and contributed to the conception and design of the work, analysis and interpretation of the data, drafting and revising the work critically for important intellectual content and final approval of the version to be published. VS contributed substantially to the conception and design, was involved in drafting and revising the work for important intellectual content and final approval of the version to be published. AW contributed substantially towards the conception and design of the work, acquisition, analysis and interpretation of data, drafting the work and revising it critically for important intellectual content and final approval of the version to be published. LK contributed substantially towards conception, design, funding application, acquisition of data, analysis, interpretation of data, drafting and revising the manuscript for important intellectual content and final approval of the version to be published. All authors read and approved the final manuscript.

## Supplementary Material

Additional file 1 Supplementary material including the detailed consenting methods, ethical approval, statistical methods and patient selection. Click here for file
