# Supplementary material for: Plasma and urine neutrophil gelatinase-associated lipocalin in the diagnosis of new onset acute kidney injury in critically ill patients
Source: Crit Care. 2014 Jul 1;18(4):R137. doi: 10.1186/cc13958 (PMC4226989; doi:10.1186/cc13958)
Supplement: Additional file 1 — Supplementary material including the detailed consenting methods, ethical approval, statistical methods and patient selection. [file cc13958-S1.doc]

Sriramajayam

**Additional File 1**

**Supplementary Material**

***Plan of Investigation***

All patients admitted to ICU were eligible to participate in this study. On admission to ICU, the patient or the consultee (if the patient was unable to consent) was consented by trained staff to participate in this study. Only patients who gave their consent were included in the study. Analysis of plasma and urine samples was withheld until consent was obtained (maximum of 96 hours) . In case of refusal to consent, the plasma and urine samples were discarded and the patient was excluded from the study. If the patient lost their capacity to consent (having consented earlier), their original decision was upheld. If a patient who was initially unable to consent (and consultee consent was obtained) decided to withdraw from the study at a later stage, their wishes were respected and they were excluded from the study.

***Duration*:**  12 months

***Data Collection***

Patients were identified by daily surveillance. Eligible patients underwent a medical record review. Data was extracted on standardised data forms. Clinical data included: demographics, co-morbidities, diuretic and/or nephrotoxin exposure, mechanical ventilation, RRT details and clinical outcomes. Clinicians were blinded to the results of NGAL values until the end of the study.

Clinical information was collected for each patient. This included demographics, co-morbidities, admission diagnosis, severity of illness score (APACHE II), RRT provision and ICU mortality at admission and during their stay in ICU. Clinical charts were reviewed for a history of exposure to potential nephrotoxins and use of diuretics in the ICU. Laboratory data was collected as part of routine patient care.

Blood and urine samples were collected from each patient at ICU admission and at 24, 48 and 72 hours of ICU stay to allow for assay of plasma and urine NGAL. Blood was only taken for clinical reasons. If a patient was discharged from the ward before the maximum of 72 hours, samples were collected for the duration of their stay in ICU only. Creatinine levels were also routinely measured on patient’s serum samples every day to calculate eGFR and determine RIFLE staging.

***Study treatment/intervention***

All patients received standard ICU care. Treatment and/or interventions were not modified due to this study. Blood and urine samples were collected as part of routine patient care and no extra blood sampling was undertaken for the study.

***Laboratory Data/ Methodology***

Blood samples were collected aseptically and processed following routine hospital and laboratory practice. Briefly, venous blood was collected using Vacutainer (Becton, Dickinson and Company UK Ltd, Oxford, UK) EDTA collection tubes and centrifuged at 4000g for 10 minutes for measurement of plasma NGAL. For measurement of creatinine, venous blood was collected using Vacutainer (Becton, Dickinson and Company UK Ltd, Oxford, UK) lithium-heparin collection tubes. Samples were centrifuged at 3200g for 10 minutes and were stored at 4ºC after routine analysis had been completed.

At the time of daily blood collection, a portion of the patient’s clinical assessment urine was reserved and sent to the laboratory and centrifuged at 4000g for 5 minutes. For plasma and urine samples, a 3 mL aliquot was frozen at -80ºC within 48 hours of collection for long-term storage until batch analysis was completed. For each patient, the ICU nurses measured total urine output. The author then calculated urine output in mL/kg/hour.

Plasma and urine samples were assayed for NGAL using Bioporto® Diagnostics immuno-turbidimetric assay on the automated clinical chemistry analyser Roche Cobas c601 (Roche Diagnostics Limited, West Sussex, UK) located in the Biochemistry Department at the Royal Berkshire Hospital. This method is currently used for research purposes only and is not in routine use for clinical specimens. Therefore, set-up and validation of the method was required.

The NGAL assay uses polystyrene particles coated with mouse monoclonal antibodies. The manufacturer’s performance claims were compared with data collected in the laboratory. Standards of NGAL were not readily available and therefore manufacturer’s internal quality control (IQC) material and patient samples were used to investigate the assay. Calibrator and IQC material were available from the manufacturer. IQC material was analysed with each batch of patient samples to ensure the assay was performing as expected. The measurement range of this assay is 25 – 5000 ng/mL (as stated by the manufacturer).

Measurement of creatinine in the serum samples were carried out using a Vitros 5.1 automated chemistry analyser (Ortho-Clinical Diagnostics, High Wycombe, UK) using standard manufacturer methods.

***Participants Inclusion and Exclusion criteria***

Inclusion:

All adult patients admitted to ICU

Exclusion:

Refusal to consent

Patients with end stage renal disease

Prior kidney transplant

Patients already on renal replacement therapy or transferred to ICU for renal replacement therapy.

Patients who meet RIFLE criteria for AKI at entry to ICU/ on first study assessment (even if not referred for this reason).

***Statistics***

Data Validation

Missing data and outlier analysis were conducted and logical error checks were conducted across all key variables. All major queries or observations regarding the validity of the data were discussed with the researchers.

Descriptive Statistics

Descriptive statistics were produced for all variables. Frequencies and proportions were reported for categorical variables. Means, standard deviations, percentiles (25%, 50%, 75%) and ranges were reported for continuous variables. Boxplots (split by AKI/no AKI, survived/didn’t survive and sepsis/no sepsis if numbers allows) were produced for urine and plasma NGAL scores on admission. Trajectory line plots, split by AKI/no AKI were produced for urine and plasma NGAL scores over time.

Characteristics of participants

Descriptive statistics for patient demographics, co-morbidities, medication including diuretic and/or nephrotoxin exposure and other key clinical variables such as APACHE II score were be produced split by AKI/no AKI. Baseline creatinine and eGFR, mortality were reported. P-values were produced for the difference between AKI and non-AKI patients for each of the reported characteristics.

Description of available data

Starting at the initial recruitment stage, the number of patients excluded from the final study analyses were reported for each stage of the study (Figure 1). In keeping with the recommendations of the STARD criteria (http://www.stard-statement.org/checklist_maintext.htm), a flowchart of the full patient selection process was produced.

Primary analysis

It is hypothesized that NGAL level is of diagnostic and prognostic value in critically ill patients in the ICU setting. Sensitivity and specificity (with 95% CIs), positive and negative likelihood ratios and predictive values of NGAL at least 24 hours before RIFLE diagnosis of AKI were calculated using BioPorto recommended thresholds (Urine NGAL 350 ng/mL., Plasma NGAL 400 ng/mL). Sensitivity and specificity (with 95% CIs), positive and negative likelihood ratios and predictive values of NGAL at least 48 hours before RIFLE diagnosis of AKI were calculated using BioPorto recommended thresholds (Urine NGAL 350 ng/mL., Plasma NGAL 400 ng/mL).

Exploratory analysis

For both plasma and urine NGAL at each time point, an ROC analysis were conducted to select a data-driven, optimal diagnostic threshold (with 95% CIs). Two by two classification matrices were reported based on these thresholds, demonstrating the performance of the test against the reference standard (RIFLE). Sensitivity and specificity, LR+ and LR-, PPV and NPV were calculated based on the selected threshold. 95% confidence intervals will be provided for all summary statistics. ROC plots for each of the three tests (at each time point) were produced to illustrate the comparative performance of the tests across different thresholds, along with the corresponding Areas under the Curve.

**Ethical Approval**

The study was approved by the National Research Ethics Committee (***Reference****:* ***Central London REC 3****.* ***11/H0716/9***) and by the Department of Research and Development, Royal Berkshire Hospital NHS Foundation Trust.
